# Burnout among Junior Nurses: The Roles of Demographic and Workplace Relationship Factors, Psychological Flexibility, and Perceived Stress

**DOI:** 10.1155/2023/9475220

**Published:** 2023-06-13

**Authors:** Yue Zhao, Xinyi Zhang, Ziling Zheng, Xiujuan Guo, Dengfeng Mou, Mi Zhao, Li Song, Nuo Huang, Jingwen Meng

**Affiliations:** ^1^Department of Nursing, Peking University First Hospital, Beijing, 100034, China; ^2^Department of Pediatrics, Peking University First Hospital, Beijing, 100034, China; ^3^Department of Nursing, Second Hospital of Shanxi Medical University, Taiyuan, 030001, China; ^4^Department of Nursing, Mingguang People's Hospital, Mingguang, 239400, China

## Abstract

**Background:**

Burnout is a common psychological syndrome among nurses, especially in the first few years of working. However, limited studies have evaluated the factors related to burnout among junior nurses.

**Aims:**

To investigate the levels of psychological flexibility, stress, and burnout among junior nurses and examine the role of demographic and workplace relationship factors, psychological flexibility, and perceived stress on burnout among junior nurses.

**Materials and Methods:**

A convenience sample of 481 junior nurses was recruited from three tertiary hospitals in China from July 2021 to August 2022. Data were collected, including demographic data, workplace relationships, psychological flexibility (measured by experiential avoidance and cognitive fusion), perceived stress, and burnout. The Independent *t*-test, one-way ANOVA, Wilcoxon–Mann–Whitney test, Kruskal–Wallis test, Spearman correlation, and hierarchical regression were used to analyze the data.

**Results:**

Junior nurses had a high level of burnout in different dimensions. Nurses with an undergraduate education, nurses working in the first year, and a lack of support from supervisors and poor colleague (nurse-nurse and nurse-doctor) relationships had the lowest level of psychological flexibility and the highest level of perceived stress and burnout. Spearman correlation indicated that experiential avoidance, cognitive fusion, perceived stress, and burnout were positively associated with each other. The regression model showed that psychological flexibility (especially cognitive fusion) and perceived stress influenced burnout in junior nurses.

**Conclusions:**

Higher psychological flexibility and lower perceived stress appear to improve burnout in junior nurses. Therefore, interventions targeting these two factors may provide a viable direction for the reduction of burnout among junior nurses. *Implications for Nursing Management*. Hospital authorities should create a harmonious working environment and provide some psychology training programs for junior nurses.

## 1. Introduction

Burnout, as an occupational hazard, has received considerable research attention in nursing over the past few years [1]. While there is no complete consensus on how to define burnout, the classic structure remains to be Maslach et al.'s description of burnout as a three-dimensional model characterized by emotional exhaustion, depersonalization, and decreased personal achievement [2]. In primary care nursing, a meta-analysis showed a prevalence of 28%, 15%, and 31% for high emotional exhaustion, high depersonalization, and low personal achievement [3]. The high prevalence of burnout among nursing professionals is unquestionable across different countries [4–7]. Lots of evidence have shown that burnout is often accompanied by anxiety, depression, and other negative emotions [8]. High levels of burnout reduce the quality of care and patient satisfaction, increase the rate of medical errors, and affect clinical nursing outcomes [1, 5, 9]. Meanwhile, it prompts more nurses to abandon the medical field, which may further exacerbate the current global shortage of nurses [10].

Junior nurses make up a large percentage of nurses and are important human resource reserves. However, studies show that young and inexperienced nurses are more likely to suffer from burnout [11], and most nurses leave in the early stage of their careers [12]. Junior nurses in China usually refer to nurses who have worked in clinical settings for less than three years after graduating from nursing school or university [13]. Junior nurses need to undergo standardized training to complete the transition to qualified nurses. The standardized training process includes rotation among different departments, such as internal medicine, surgery, obstetrics and gynecology, pediatrics, and emergency and intensive care unit, as well as regular and irregular theoretical and operational examinations [14]. The transition is difficult owing to unfamiliar working environments, different professional skills, and interpersonal relationship adaptation, which are significant physical and psychological challenges for junior nurses [15]. Over time, these challenges unconsciously become catalysts for their burnout and even resignation [16].

Burnout is a complex multifactorial problem that is not easily addressed, even among nurses. Individual-specific factors (such as demographic variables, stress, psychological flexibility, personality, and coping strategies) and existing workplace factors (like job demands, job control, and organizational support) have been found to have varying degrees of relationships with burnout levels in numerous studies [17–21].

The relationship between stress and burnout is obvious, both from a psychological and biological perspective [22, 23]. Generally, burnout is believed to be an extreme reaction when individuals cannot smoothly cope with work stress, and it is a state of exhausted emotions, attitudes, and behaviors caused by individuals under long-term work pressure [24]. The effects of chronic stress on the functioning of biological processes in an organism can affect psychological processes and social behavior. Thus, according to clinical psychologists, burnout is not necessarily related to work but to stress [25]. Results from a cross-sectional study of 799 nurses showed that stress indirectly affected burnout through perceived social support and psychological capital [26]. In addition, a cross-sectional survey of new graduate nurses revealed that 76% of participants reported moderate-to-severe stress [27]. While some stress can be helpful, overwhelming stress can lead to poor performance at work and serious illnesses such as high blood pressure, depression, and sleep disorders [28].

Psychological flexibility was defined as “the ability to more fully engage with the present moment as a conscious person and to change or persist in behavior in the service of a valued goal” [28]. Psychological inflexibility is the opposite of that and mainly includes two important aspects: experiential avoidance and cognitive fusion [29]. In short, experiential avoidance is the tendency for individuals to avoid or escape from some unpleasant internal mood, which could lead to psychological distress and ineffective behaviors. A higher degree of experiential avoidance is associated with more emotional distress and lower life function, thus reducing work performance [30]. Cognitive fusion refers to the domination of thinking in behavioral regulation over other available processes [31]. Cognitive fusion can enhance an individual's believability of negative thoughts, leading to emotional discomfort [32]. Studies have shown that the lower the psychological flexibility was, the more the clinical nurses were tired of their job [20]. Meanwhile, the literature indicated a direct and predictive relationship between psychological inflexibility and burnout among nurses in the acute phase of the epidemic [33].

Besides, organizational factors have consistently been implicated among the complex determinants of burnout. In a recent report, burnout was considered as a result of an imbalance between job demands and resources [34]. The resources of clinicians include tangible and intangible resources in the work environment, such as meaning in work, job control, and social support from peers and supervisors. Multiple studies have shown that, in addition to increasing the number of nurses, changing the characteristics of the work environment, including training support, positive physician-nurse relationships, nurse autonomy, and support from supervisors, can also increase job satisfaction and reduce burnout in nurses [35–37].

The retention and successful transition of junior nurses is one of the guarantees for the future stable development of health care [38], while burnout is a stumbling block. It is urgent to find ways to retain them, but there are few studies on burnout among junior nurses. Therefore, we aimed to investigate the levels of psychological flexibility, stress, and burnout among junior nurses in different sociodemographic and workplace relationships. Furthermore, we sought to explore the influence of these individual and organizational factors on burnout among junior nurses.

## 2. Materials and Methods

### 2.1. Setting, Sample, and Data Collection

A cross-sectional design was used in this study. Participants were nurses recruited online from three tertiary hospitals in China from July 2021 to August 2022. The inclusion criteria for all participants were as follows: (1) aged 18 years or older; (2) with at most three years of work experience; and (3) provided informed consent. The exclusion criteria included nurses with psychological disease or who were taking a long leave of absence (e.g., maternity leave).

The sample size was calculated using G^*∗*^Power 3.1.9.7. In the multiple linear regression, a fixed model and *R*^2^ deviation from zero were selected as the statistical method. The statistical significance level was set at *α* = 0.05, with an effect size of 0.1 and a statistical power (1 − *β*) of 0.95, considering a total of 12 predictors. Theoretically, a minimum sample size of 270 was calculated.

With the assistance of the department of nursing, the call to participate was sent to 526 targeted nurses by link via the WeChat platform. The nurses decided whether to participate after reading the purpose and content of the study and had the right to withdraw at any time. Researchers checked the platform daily and stopped data collection once it reached the target. Finally, we collected 481 valid questionnaires (response rate: 91.4%) through the Wenjuanxing platform (https://www.wjx.cn/). All questions were set as mandatory, so there were no missing data.

### 2.2. Measures

#### 2.2.1. Demographic and Workplace Factors

Demographic data were collected, including age, gender, education level, salary, and working experience. Workplace relationships included the relationship between nurses, the relationship between nurses and doctors, and the support received from supervisors.

#### 2.2.2. Psychological Flexibility

*(1) Experiential Avoidance*. The Acceptance and Action Questionnaire II (AAQ-II), a 7-item instrument, was used to assess the level of experiential avoidance [30]. The AAQ-II is a 7-point Likert scale from 1 (never) to 7 (always). A higher score indicates a higher level of experiential avoidance. The minimum and maximum total score of the scale is 7 and 49, respectively. Cronbach's *α* coefficient of the Chinese version of AAQ-II was 0.88 [39]. Cronbach's *α* of the scale was 0.95 in this study.

*(2) Cognitive Fusion*. The Cognitive Fusion Questionnaire-Fusion (CFQ-F), a 9-item instrument assessing personal cognitive fusion, was developed by Gillanders et al. [40]. Items are rated on a 7-point Likert scale from 1 (never) to 7 (always). The possible score can range from 9 to 63, with higher scores denoting more serious cognitive fusion. Cronbach's *α* coefficient of the Chinese version of CFQ-F was 0.92 [41]. Cronbach's *α* of the scale was 0.96 in this study.

#### 2.2.3. Perceived Stress

The level of perceived stress was assessed with the Chinese Perceived Stress Scale (CPSS) [42], which was revised from the Perceived Stress Scale (PSS) [43]. The CPSS consists of a total of 14 items, with each item rated on a 5-point Likert scale from 0 (never) to 4 (very often). The total score of the CPSS scale ranges from 0 to 56, and a higher score indicates a greater level of perceived stress. Cronbach's *α* coefficient of the CPSS was 0.84 [26]. In this study, Cronbach's *α* of the scale was 0.70.

#### 2.2.4. Burnout

Burnout was measured using the Chinese version of the Maslach Burnout Inventory: Human Services Survey for Medical Personnel (MBI-HSS-MP) [44, 45]. It consists of a total of 22 items across three dimensions of emotional exhaustion, depersonalization, and personal accomplishment. Each item is scored on a 7-point Likert scale from 0 (never) to 6 (every day). Scores of 19–26 or ≥27 on emotional exhaustion, 6–9 or ≥10 on depersonalization, and 34–39 or ≤33 on personal accomplishment were indicative of moderate or high burnout for the respective dimensions. For the sake of consistency of interpretation, the items of personal accomplishment were reverse-scored and renamed as low personal accomplishment when calculating the total score. The total score of the MBI ranges from 0 to 132, and a higher score denotes more severe job burnout. Cronbach's *α* coefficient for the Chinese version of MBI was 0.62 [45]. Cronbach's *α* of the scale was 0.80 in this study.

### 2.3. Data Analysis

Data analysis was performed using IBM SPSS Statistics, version 25 (IBM Corp., Armonk, NY, USA). Firstly, descriptive data were generated for participants' overall characteristics. Then, based on the normality test, we used the independent *t*-test or one-way analysis of variance (ANOVA) to compare levels of perceived stress and burnout, and the Wilcoxon–Mann–Whitney test or Kruskal–Wallis test was used to compare levels of experiential avoidance and cognitive fusion between nurses with different demographic and workplace relationship characteristics. The relationship between these psychological measurements was examined using the Spearman correlation. Finally, hierarchical regression analysis was used to construct models to assess the individual strength of different variables affecting burnout and how much the models explained burnout.

### 2.4. Ethical Considerations

Ethical approval for this study was obtained from the Clinical Research Ethics Committee of Peking University First Hospital (approval no. 2021-415). Informed consent was obtained voluntarily from all participants, and all the data were recorded and analyzed anonymously.

## 3. Results

### 3.1. Descriptive Statistics

Table 1 shows the descriptive statistics for participants' demographic, workplace, and psychological characteristics. Of the 481 nurses who completed the questionnaire, most were female (86.7%), with a mean age of 24.2 years. Approximately half of the participants held undergraduate education, while only 10.2% had postgraduate degrees. In total, 42% of the participants had only one year of work experience, and nearly 90% had a monthly salary of less than ¥5000. When asked about social relationships and support in the workplace, the vast majority of respondents reported being satisfied with coworker relationships and supervisor support, while about one-quarter of the respondents reported poor relationships between doctors and nurses. The participants' overall scores of experiential avoidance, cognitive fusion, perceived stress, and burnout are shown in Table 1. The scores for each of the three dimensions of burnout suggested high levels of emotional exhaustion (mean = 33.48, SD = 6.53) and depersonalization (mean = 16.07, SD = 5.23) and low level of personal achievement (mean = 20.75, SD = 4.51), indicating high burnout.

### 3.2. Comparisons of Junior Nurses' Psychological Flexibility, Perceived Stress, and Burnout between Groups

Significant differences in the score of several scales were found according to participants' demographic characteristics and workplace factors (Table 2). The preliminary analysis showed that female nurses had significantly higher levels of experiential avoidance. Nurses with a junior college education had significantly lower levels of experiential avoidance, cognitive fusion, and burnout than those with undergraduate education. There was little difference between undergraduate and postgraduate nurses, although undergraduate nurses scored slightly higher on perceived stress and burnout than the other two groups. Additionally, there was a decreasing trend in the scores of all four scales with longer work years in new nurses. Perceived stress decreased significantly across all years of work experience, while the improvement in the other three aspects did not reach statistical differences until the third year. Moreover, self-reported data showed that poor relationships and support from colleagues or supervisors were accompanied by significant psychological inflexibility, stress, and burnout.

### 3.3. Correlations between Psychological Flexibility, Perceived Stress, and Burnout

The results of the correlational analysis between four psychological measurements are presented in Table 3. Overall, experiential avoidance, cognitive fusion, perceived stress, and burnout were positively associated with each other. For each dimension of burnout, the largest correlations observed were between depersonalization and other measures. The correlation between accomplishment and cognitive fusion was relatively weak, while that between accomplishment and experiential avoidance failed to reach statistical significance.

### 3.4. The Roles of Demographics and Workplace Relationship Factors, Psychological Flexibility, and Perceived Stress in Burnout among Junior Nurses

Table 4 displays the results of the hierarchical regression analysis. As can be seen from the table, demographics and workplace relationship factors were entered in the first step, psychological flexibility in the second, and perceived stress in the third. Working experience, the relationship between nurses, the relationship between doctors and nurses, and supervisor support were significantly associated with burnout in the first step, while none of these remained statistically significant after psychological flexibility was added to the model. By comparing models 2 and 3, it can be seen that the standardized beta coefficients of experiential avoidance reduced to no statistical significance when considering perceived stress. Moreover, the adjusted *R*^2^ of each regression indicated that the three sequential models explained 9.9%, 35.2%, and 43.9% of the variance in burnout, respectively.

## 4. Discussion

Although burnout has been noted in nurses for years, few studies have focused specifically on junior nurses. Our study examined the role of demographic and workplace relationships, psychological flexibility, and perceived stress on burnout and reported levels of these psychological indicators among junior nurses.

Our findings indicated a high level of burnout in different dimensions in our sample, which confirms that young nurses are at high risk of burnout. The COVID-19 pandemic had significant impacts on nurses' mental health [27, 46, 47]. China was at a lull between outbreaks during data collection for this study, and there were only a few COVID-19 cases in the sample hospitals. However, the workload associated with the “dynamic zero-COVID policy” and demands put on hospital staff (e.g., frequent nucleic acid testing, strict epidemic prevention and control measures, and restricted mobility) may have increased burnout. Xie et al.'s cross-sectional study of newly graduated nurses from 13 provinces in China before the pandemic focused on nurses with less than three years of experience and reported less severe burnout than our findings [48]. Province, hospital levels, and epidemic status may primarily explain the differences. Moreover, our study demonstrated that several demographic and workplace relationships are associated with these variables. We found that nurses with a junior college education had the lowest level of psychological inflexibility, perceived stress, and burnout, while nurses with an undergraduate education had the worst scores among the three educational groups. This is partially inconsistent with previous studies, which showed that nurses with higher levels of education had lower levels of burnout [49, 50]. The reasons for the difference are unclear, given that education is confounded by other variables such as responsibilities, resources, and personal attributes [34]. For our findings, it is possible that junior college nurses have fewer responsibilities and encroachment on personal time. In addition, postgraduate nurses may have boosted their strategies to deal with adversity during education and may get more support and resource at work, while undergraduate nurses are more likely to experience a mismatch in job demands and resources, leading to stress and burnout. Future qualitative studies are needed to delineate the reasons behind this finding.

We also found that junior nurses with longer years of tenure had higher psychological flexibility and lower levels of stress and burnout, similar to the findings of earlier studies [21]. This finding can be explained by capacity and maturity gained from work or life experience, although it may also be due to survival bias. Longitudinal studies are needed to eliminate the bias caused by resignation and support the idea that experience can improve psychological flexibility in the absence of additional psychoeducation. Interestingly, perceived stress declined significantly across each additional year of work experience, while the improvement in burnout was not so rapid. The finding suggests that the accumulation of work experience can reduce stress caused by a lack of professional knowledge and skills, while the contributors of burnout may be more complex and require additional attention.

In the univariate analysis as well as the first model of hierarchical regression, our results showed a significant association between workplace relationships and nurse burnout. However, these effects were no longer statistically significant when psychological flexibility was entered into the model. This appears to be contrary to other studies and reports of burnout among health workers [34, 51]. One likely reason is that we used subjective questions rather than objective scales when assessing workplace relationships; thus, the responses obtained highly depend on subjective perceptions. In addition, some aspects of psychological flexibility, such as cognitive integration, may distort or reinforce the individual's perception of external evaluations to some extent. The relationship between workplace relationships and psychological flexibility in Table 2 supports this inference. Social support from coworkers and supervisors has been reported to reduce stress reactions and promote personal meaningfulness at work [52, 53]. The perception of relationships and support at work could increase nurses' confidence and alleviate adverse emotions by changing the process of cognitive appraisal [54]. The individual's psychological flexibility may have a similar effect in the process.

Our finding of a negative association between psychological flexibility and burnout corroborates with many other studies among nurses and nursing students [21, 33, 55, 56]. In addition, consistent with the concept of burnout as an outcome of chronic stress [57], the effect of perceived stress was reflected in the final model. Notably, psychological flexibility had an independent effect on burnout, suggesting that some aspects of psychological flexibility can directly affect burnout instead of indirectly through stress reduction. An imbalance between job demands and resources is considered to be the reason leading to stress and burnout [58, 59]. Psychological flexibility, as an intangible personal resource, helps individuals adhere to value-based actions in the presence of uncomfortable feelings [60]. The positive feedback that comes with effective work will guarantee that individuals are engaged in their work, realize their value in the workplace, and ultimately reduce burnout [61].

A strength of this study is that it was a multicenter study that focused exclusively on junior nurses. In addition, we considered both organizational and individual factors. However, the study has some limitations. Due to the cross-sectional design of the study, it is impossible to determine the causality between the variables. Furthermore, workplace relationships were evaluated using self-designed questions, which may reduce the objectivity of these factors. Nevertheless, this, in turn, suggests that stress and burnout resulting from perceived workplace relationships may be modifiable through specific processes of psychological flexibility.

Future researchers should carry out longitudinal and qualitative studies to clarify the different processes of burnout and how psychological flexibility regulates these processes. Path analysis would help assess the causal relationship and potential moderators. In addition, future studies should explore the possibility of providing extensive training for junior nurses to improve their psychological flexibility, such as mindfulness and acceptance commitment therapy.

### 4.1. Implications for Nursing Management

Junior nurses' job burnout will affect their professional identity, professional ability, and career development. In order to prevent early turnover and job change, it is necessary for hospital authorities to help junior nurses improve their burnout levels. We recommend that nurse managers prioritize improving organizational culture and enhancing the mental health of their employees, which may include creating a fair, non-blaming, supportive working environment; sharing tips for junior nurses to quickly acquire skills and deal with relationships in a new department; and providing some psychology training focused on psychological flexibility, stress, and burnout, such as mindfulness and acceptance and commitment therapy.

## 5. Conclusions

The results of this study corroborate that new nurses are at a high risk of burnout. Workplace relationships, psychological flexibility, and perceived stress impact burnout, while the latter two may be more fundamental.

## Figures and Tables

**Table 1:** Demographic, workplace, and psychological measurement characteristics of the participants (*N* = 481).

Variable	*N*	%
Gender		
Female	420	87.3
Male	61	12.7
Education		
Postgraduate	49	10.2
Undergraduate	248	51.6
Junior college	184	38.3
Salary (yuan/m)		
≤5000	281	58.4
5001∼10000	156	32.4
≥10000	44	9.1
Work experience		
1 year	204	42.4
2 years	164	34.1
3 years	113	23.5
The relationship between nurses		
Good	462	96.0
Poor	19	4.0
The relationship between doctors and nurses		
Good	370	76.9
Poor	111	23.1
Supervisor support		
Good	442	91.9
Poor	39	8.1
	M	SD
Age (years)	24.21	1.88
Experiential avoidance	31.70	9.61
Cognitive fusion	43.19	12.19
Perceived stress	25.50	6.44
Burnout	76.80	11.56

M = mean; SD = standard deviation.

**Table 2:** Differences in nurses' experiential avoidance, cognitive fusion, perceived stress, and burnout by demographic characteristics and workplace relationship factors (*N* = 481).

Variable	*N*	Experiential avoidance		Cognitive fusion		Perceived stress		Burnout	
		M (IQR)	*z/H* (*p*)	M (IQR)	*z/H* (*p*)	M (SD)	*t/F* (*p*)	M (SD)	*t/F* (*p*)
Gender			−2.232 (0.026)^*∗*^		−1.558 (0.119)		1.824 (0.069)		1.723 (0.086)
Female	420	33.00 (15.75)		45.00 (25.00)		25.70 (6.55)		77.15 (11.49)	
Male	61	28.00 (21.00)		42.00 (27.00)		24.10 (5.51)		74.43 (11.89)	
Education			18.872 (<0.001)^*∗∗∗*^		17.842 (<0.001)^*∗∗∗*^		2.709 (0.068)		4.859 (0.008)^*∗∗*^
Postgraduate	49	35.00 (11.00)^c^		47.00 (17.50)		25.98 (6.64)		76.69 (11.40)	
Undergraduate	248	35.00 (14.00)^c^		47.50 (22.50)^c^		26.05 (5.95)		78.30 (11.54)^c^	
Junior college	184	28.00 (17.75)^a,b^		39.50 (24.00)^b^		24.64 (6.95)		74.82 (11.39)^b^	
Salary (yuan/m)			1.942 (0.379)		3.845 (0.146)		0.306 (0.737)		1.143 (0.320)
≤5000	281	31.00 (18.00)		44.00 (27.00)		25.32 (6.63)		77.08 (11.37)	
5001∼10000	156	33.50 (14.00)		45.00 (23.00)		25.69 (6.15)		75.82 (11.54)	
≥10000	44	35.00 (12.00)		51.00 (12.75)		26.00 (6.33)		78.55 (12.78)	
Work experience			14.934 (0.001)^*∗∗*^		10.659 (0.005)^*∗∗*^		34.370 (<0.001)^*∗∗∗*^		6.258 (0.002)^*∗∗*^
One year	204	34.50 (13.75)^c^		47.00 (23.00)^c^		27.85 (6.79)^b,c^		78.57 (11.8)^c^	
Two years	164	34.50 (17.50)^c^		45.00 (22.50)^c^		24.95 (5.28)^a,c^		76.66 (11.65)	
Three years	113	28.00 (16.50)^a,b^		39.00 (24.00)^a,b^		22.07 (5.61)^a,b^		73.83 (10.41)^a^	
The relationship between nurses			−3.565 (<0.001)^*∗∗∗*^		−3.048 (0.002)^*∗∗*^		−3.851 (<0.001)^*∗∗∗*^		−4.089 (<0.001)^*∗∗∗*^
Good	462	32.00 (16.00)		45.00 (25.00)		25.27 (6.24)		76.37 (11.47)	
Poor	19	41.00 (10.00)		54.00 (20.00)		31.00 (8.60)		87.26 (8.70)	
The relationship between doctors and nurses			−5.447 (<0.001)^*∗∗∗*^		−5.805 (<0.001)^*∗∗∗*^		−5.199 (<0.001)^*∗∗∗*^		−4.344 (<0.001)^*∗∗∗*^
Good	370	31.50 (15.00)		42.50 (25.00)		24.69 (6.32)		75.57 (11.60)	
Poor	111	38.00 (12.00)		53.00 (12.00)		28.22 (6.13)		80.91 (10.49)	
Supervisor support			−5.160 (<0.001)^*∗∗∗*^		−4.979 (<0.001)^*∗∗∗*^		−5.573 (<0.001)^*∗∗∗*^		−5.170 (<0.001)^*∗∗∗*^
Good	442	31.00 (17.00)		44.00 (25.00)		25.03 (6.35)		76.02 (11.27)	
Poor	39	39.00 (8.00)		54.00 (14.00)		30.85 (4.96)		85.74 (11.22)	

M = mean/median; SD = standard deviation; IQR = interquartile range; ^*∗*^*p* < 0.05; ^*∗∗*^*p* < 0.01; ^*∗∗∗*^*p* < 0.001. ^a^Significant difference with those at postgraduate level or one-year work experience after Bonferroni adjustments. ^b^Significant difference with those at undergraduate level or two-year work experience after Bonferroni adjustments. ^c^Significant difference with those at junior college level or three-year work experience after Bonferroni adjustments.

**Table 3:** Spearman's correlations between burnout, perceived stress, experiential avoidance, and cognitive fusion (*N* = 481).

Variables	Burnout	Exhaustion	Depersonalization	Low personal accomplishment	Perceived stress	Experiential avoidance	Cognitive fusion
Burnout	1.000						
Exhaustion	0.834^*∗∗*^	1.000					
Depersonalization	0.789^*∗∗*^	0.576^*∗∗*^	1.000				
Low personal accomplishment	0.411^*∗∗*^	0.037	0.038	1.000			
Perceived stress	0.640^*∗∗*^	0.551^*∗∗*^	0.565^*∗∗*^	0.228^*∗∗*^	1.000		
Experiential avoidance	0.553^*∗∗*^	0.490^*∗∗*^	0.562^*∗∗*^	0.066	0.634^*∗∗*^	1.000	
Cognitive fusion	0.591^*∗∗*^	0.515^*∗∗*^	0.588^*∗∗*^	0.097^*∗*^	0.660^*∗∗*^	0.840^*∗∗*^	1.000

^*∗*^ *p* < 0.05; ^*∗∗*^*p* < 0.01.

**Table 4:** Hierarchical multiple regression models in explaining nurses' burnout (*N* = 481).

Variables	Model 1		Model 2		Model 3	
	*B* (SE)	*β* (*p*)	*B* (SE)	*β* (*p*)	*B* (SE)	*β* (*p*)
Independent variable						
Age	0.327 (0.390)	0.053 (0.402)	−0.043 (0.333)	−0.007 (0.897)	−0.142 (0.310)	−0.023 (0.646)
Gender (ref: female)	−2.297 (1.508)	−0.066 (0.128)	−0.999 (1.287)	−0.029 (0.438)	−0.658 (1.197)	−0.019 (0.583)
Postgraduate (ref: undergraduate)	−2.289 (2.146)	−0.060 (0.278)	−0.992 (1.831)	−0.026 (0.588)	−0.673 (1.703)	−0.018 (0.693)
Junior college (ref: undergraduate)	−1.946 (1.216)	−0.082 (0.110)	−0.593 (1.038)	−0.025 (0.568)	−1.366 (0.970)	−0.057 (0.159)
Salary (yuan/m)	−0.949 (0.812)	−0.054 (0.243)	−1.039 (0.690)	−0.059 (0.133)	−0.787 (0.642)	−0.045 (0.221)
Working experience	−1.986 (0.716)	**−0.136 (0.006)**	−0.946 (0.612)	−0.065 (0123)	0.754 (0.603)	0.052 (0.212)
The relationship between nurses (ref: poor)	−5.662 (2.766)	**−0.095 (0.041)**	−4.484 (2.351)	−0.076 (0.057)	−3.683 (2.188)	−0.062 (0.093)
The relationship between doctors and nurses (ref: poor)	−3.182 (1.255)	**−0.116 (0.012)**	−0.222 (1.087)	0.008 (0.838)	0.145 (1.012)	0.005 (0.886)
Supervisor support (ref: poor)	−6.681 (1.965)	**−0.158 (0.001)**	−3.088 (1.688)	−0.073 (0.068)	−1.725 (1.578)	−0.041 (0.275)
Experiential avoidance			0.206 (0.080)	**0.171 (0.011)**	0.082 (0.076)	0.068 (0.281)
Cognitive fusion			0.373 (0.063)	**0.393 (<0.001)**	0.224 (0.061)	**0.236 (<0.001)**
Perceived stress					0.755 (0.088)	**0.421 (<0.001)**
Model diagnosis and fit						
*F* value (*p* value)	6.873 (<0.001)		24.655 (<0.001)		32.334 (<0.001)	
Variance inflation factor	1.006–2.147		1.018–3.311		1.019–3.567	
*R*^2^ (adjusted *R*^2^)	0.116 (0.099)		0.366 (0.352)		0.453 (0.439)	
∆*R*^2^	0.116		0.250		0.087	

*B* *=* unstandardized regression coefficient; SE = standard error; *β* *=* standardized regression coefficient; significant values are reported in bold.

## Data Availability

The data used to support the findings of this study are available from the corresponding author upon request.
